# Weight restoration in adolescent anorexia: parents’ goal-directed processes

**DOI:** 10.1186/s40337-022-00676-w

**Published:** 2022-12-07

**Authors:** Krista D. Socholotiuk, Richard A. Young

**Affiliations:** 1 Faculty of Education, Simon Fraser University, 8888 University Drive, Burnaby, BC V5A 1S6 Canada; 2 Department of Educational and Counselling Psychology, and Special Education, University of British Columbia, 2125 Main Mall, Vancouver, BC V6T 1Z4 Canada

**Keywords:** Family-based treatment, Parent action, Weight restoration, Qualitative

## Abstract

**Background:**

Parent-led weight restoration is a key intervention of family-based treatment, an empirically supported treatment for adolescent anorexia. Successful outcomes in family-based treatment depend almost entirely on parental action, yet current understandings of this intervention are primarily informed by professional theory and expert perspectives. Comparatively little is known about parents’ goals and actions while implementing the treatment, despite goal-directed action being an explicit framework of family-based treatment. This study seeks to investigate parents’ involvement in weight restoration from the perspective of the goal-directed actions they construct and engage in themselves and with others. This study focuses on the phenomenon of parent-led weight restoration as a project and addresses the following research question: “How do parents participate in the weight restoration of their adolescent as the adolescent recovers from anorexia nervosa?”.

**Method:**

This multicase study used the action project method and conceptual framework of contextual action theory to examine four cases of five parents engaged in actions to help their adolescent regain weight and recover from anorexia. Data were collected using multi-part interviews and analyzed according to the action project method and the multicase approach.

**Results:**

Parents’ weight restoration projects were identified and grouped based on three common a themes. The primary theme, progressing toward health and well-being, was supported by three key processes: maintaining a holistic focus, trusting, and monitoring progress. Two secondary themes captured actions that were integral to the parents’ projects, but with less prominence. Secondary themes were creating capacity, which was supported by three processes (managing emotions to maintain a helpful focus, personal work, and resourcing time and finances), and coordinating and negotiating partnerships. The socio-cultural valuing of the thin ideal emerged as a unique process salient in one case. This study presents a goal-directed and contextual perspective on how parents translated the principles of family-based treatment into their daily lives. It joins a small but growing body of work concerned with generating new understandings and frameworks for practitioners and researchers to enhance the effectiveness of family-based treatment in community settings.

## Plain English summary

Family-based treatment is one of the leading treatments for helping adolescents recover from anorexia. Parents play an essential role in this treatment, yet little is known about how parents translate the principles of FBT into day-to-day life. We studied four cases of five parents while they were using FBT to help their adolescent recover from anorexia. Overall, we found these parents’ actions were guided by three common and overarching goals. First, parents were focused on progressing toward health and well-being, using processes like keeping a focus on adolescent development, trusting, and monitoring progress. Second, parents were focused on creating psychological, emotional, and financial capacity to be successful in FBT, where many of their actions were shaped and constrained by the resources available to them. Finally, parents were focused on negotiating and coordinating partnerships with their co-parent (primarily), as well as others, to stay united in their efforts.

## Background

Family-Based Treatment [1, FBT] is one of the most researched treatment interventions for adolescent anorexia [2, 3]. The approach has three phases, and parents play an essential role throughout. In the first phase, parents are assigned responsibility for their adolescent’s eating and food-related decisions until they can eat well and gain weight independently [1]. It is presumed adolescents will strenuously oppose parent-led weight restoration, and that parents might feel trepidation about its implementation. Accordingly, motivating parents to remain confident and committed to the treatment is an important feature of treatment [1, 4]. FBT holds several theoretical positions to help parents remained focused on this goal. For example, its non-aetiological stance is designed to absolve parental guilt that might introduce self-doubt and undermine parental self-efficacy [1]. Similarity, normal adolescent development is assumed to have been stopped by anorexia to help parents accept the need to temporarily take charge. Other family problems or adolescent developmental issues are postponed until weight is regained, and only addressed insofar as it relates to the task of ensuring steady and ongoing maintenance of weight. In Phases II and III, the central therapeutic activity is for parents to gradually transfer control of food and weight back the adolescent by phasing out their management and supervision of the adolescent’s meals and eating disorder behaviors.

Under conditions of high internal validity, up to 40% of adolescents treated with FBT achieve favorable outcomes on percentage of ideal body weight and eating disorder symptom scales [5]. Further, accumulated evidence shows FBT is tenable outside of academic and hospital settings [6–9]. Some adolescents report weight restoration was initially unwanted but acceptable in retrospect, taking pressure off and providing a sense of being cared for, amongst other things [10]. Some parents have found treatment to initially be a container or map [10, 11] bringing elements of positive change for the families for whom it works [10, 12]. Yet, there remains much variability in outcomes for adolescents and their families [11, 13]; many do not complete treatment, and the effectiveness of the treatment remains uncertain for a sizeable proportion of families [5].

Recommendations for improving outcomes for families have focused on improving practitioner fidelity to the manual [14], such as better training and clinical self-efficacy with the interventions [15, 16], as well as treatment augmentations that target parents’ self-efficacy with the core elements of FBT [17–19]. Other studies have sought to identify family patterns or parental traits that might be predictive of response and change processes in FBT, such as parental intolerance of uncertainty [20], parental attachment and mentalization [21], family functioning and relationship quality [22].

The success of FBT rests almost entirely on parental action, yet the actions of parents and how they engage with FBT is most often captured indirectly through professional perceptions of parents’ behaviors [23–25], survey techniques [26, 27], or parents’ retrospective accounts [11, 13, 28]. The case has been made elsewhere for goal-directed action as a significant framework for therapy [29], based in part on the implicit prospective nature of therapy and its goal-directed focus. Goal-directed action is an explicit framework in FBT, and its three phases are described as a distinct sequence of goals designed to keep the therapist and family moving toward the future goal of recovery [1]. Given the goal-directed focus in FBT, and that success in FBT rests almost entirely on parent actions, it is surprising that no research has attended to parents’ goals and actions while participating in the treatment.

A variety of action theorists [30–33] note human agency and the social embeddedness of behavior requires explanatory models that consider context and can take into account the statements people make about their reasons and intentions for doing things. Contextual action theory [34, 35] is a conceptual and methodological approach to understanding and explaining human behavior. It looks to the goal of a person’s action, as well as antecedent conditions and functional explanations, for understanding. That is, it looks at human behavior (action) and asks how it is understood by the person enacting it in the unique context in which it is situated. Contextual action theory assumes that when actions coalesce around meaningful goals that endure over time, they yield projects (short-term) and careers (long-term). For example, in FBT, a parent’s provision of meal support, participation in treatment, and coordinating new work routines can be considered weight restoration actions. A weight restoration project is constituted when the parent enacts actions serving the goal of weight regain and recovery over a period of weeks and months [36]. In turn, this weight restoration project may serve the larger goal of raising a healthy adolescent, indicative of a parenting career.

The purpose of this study is to investigate parents’ involvement in weight restoration from the perspective of the goal-directed actions they construct and engage in themselves and with others. This study focuses on the phenomenon of parent-led weight restoration as a project and addresses the following research question: “How do parents participate in the weight restoration of their adolescent as the adolescent recovers from anorexia nervosa?”.

## Method

The qualitative action-project method [37, 38] was used to collect and analyze data. Contextual action theory [34] provided the conceptual and methodological framework, and the research strategy was Stake’s [39] multicase study approach. In this approach, action is the unit of analysis, and it is viewed as being composed of goals, internal cognitive and emotional processes, and observable behaviors. No a priori assumptions were made about the content of actions or the types of goals or projects that parents would construct.

### Participants

Five parent participants (three mothers, one father-mother dyad) of 4 adolescents between 13 and 16 years of age (*M*_*age*_ = 14.5; *SD* = 1.73) were recruited for this study in Vancouver, Canada. The original data set included six parents, but one case was removed. While this parent was active in supporting their adolescent’s weight regain, the treatment team had made many departures from FBT, including asking the parent to leave appointment scheduling to the adolescent. Table 1 summarizes the demographic information for the remaining cases. In three cases, participants responded to advertisements at local child and youth mental health centres; in one case, the parent responded to an on-line advertisement. During the initial telephone screening, participants were asked questions relevant to the inclusion criteria, which included being a parent of an adolescent (between 11 and 19 years of age) actively engaged in community-based treatment for anorexia using FBT. Adolescents could be in the early, middle, or late stages of weight restoration, and consistent with multicase approach, the study was open to including model and atypical cases in recognition that seeking out and portraying different perspectives, including contradictions or competing values, would help further understanding of how parents participate in weight restoration [39].

**Table 1:** Case demographic information

Case	Age (years)		Duration (months)	
	Adolescent	Parent	Weight restoration	Anorexia
1	16	54	9	16
2	13	44	12	11
3a	16	48	11	10
3b		42		
4	13	44	3.5	4
*Average*	14.50	46.40	8.87	10.25
*SD*	1.73	1.73	3.80	4.92

*SD* standard deviation

Two mothers (Cases 2 & 4) were heads of single-parent homes with shared custody of their adolescent, one mother (Case 1) was married, but her partner declined to participate in the study, and the parental dyad (Case 3) were married. Participants were born in Canada (*n* = 3) and Western European countries (*n* = 2). Four parents identified as Caucasian, one identified as Indigenous. At the first and final data gathering sessions, each parent was paid an honorarium for their participation.

### Procedures and analysis

**Fig. 1:**
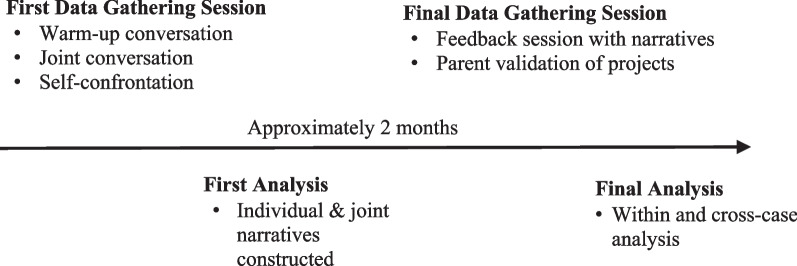
Sequence of action-project method data collection and analysis procedures for one case

Data collection and analysis unfolded in four distinct phases (Fig. 1. Sequence of action-project method data collection and analysis procedures for one case). The action-project method uses multiple procedures to access information about individual and joint actions, and sources of data gathering correspond with three perspectives on action. Specifically, video-recorded conversations provided data on the parents’ weight restoration actions and behaviors, video-playback of the conversations (i.e., self-confrontation) gave access to parents’ thoughts and emotions, and systematic analysis of both the video-recorded conversations and self-confrontation got at contextual and social meaning. All data gathering sessions were conducted at private locations convenient to the participants: community mental health offices (*n* = 3); a university research space (*n* = 1), and a community recreation centre (*n* = 1). For consistency, the first author (Socholotiuk), a doctoral student with 5 years experience as an adolescent eating disorders clinician, was involved in the data collection and analysis for all four cases. Two associate professors/psychologists and two doctoral students in counseling psychology assisted with the data collection, and the research team involved in the analysis included the first and second author and two masters’ students in counseling psychology. All members of the research team were familiar with the philosophical underpinnings of method, and all data from the data gathering sessions were recorded and transcribed verbatim.

#### First data gathering session

The first data gathering session started with a warm-up conversation with the parent (or parents, in Case 3) and two researchers. The warm-up conversation served various purposes, including establishing rapport, learning about each parent’s experience with weight restoration, and helping them to identify topics pertinent to weight restoration and their engagement with it. The warm-up conversation concluded by inviting parents to select one of the topics to discuss at greater length in the joint conversation.

The average duration of the warm-up conversation, as well as subsequent data gathering sessions, is presented in Table 2.

**Table 2:** Duration (minutes/seconds) of data gathering sessions

Case	First data gathering session			Final data gathering session	Total
	Warm-up interview	Joint conversation	Self-confrontation		
1	23:35	23:00	2:23:43	1:16:40	4:26:58
2	22:27	16:27	51:09	33:17	2:03:20
3a	37:39	17:02	45:58	39:27^a^	2:20:06
3b			58:21		2:32:29
4	20:43	18:16	57:14	38:31	2:14:44
Total	1:44:24	1:14:45	5:56:25	3:07:55	13:37:37
*Average*	26:06	18:41	1:11:17	46:59	2:43:31
*SD*	7:47	2:58	40:48	19:59	58:46

*3a* mother; *3b* father; *SD* standard deviation

^a^This time reflects the total duration of the parental dyad’s final data gathering session. Within this period of time, each parent separately reviewed their individual narratives with a researcher: mother = 5:32 min.; father = 7:25 min

For the joint conversation in Case 3, the researchers left the room, allowing the parents to self-direct the conversation. For cases with an individual parent participant, a researcher served as the parent’s conversation partner. The procedures of the action-project method are designed to produce rich description of dyadic action but are flexible to allow for adaptations provided the research problem is framed from the perspective of goal-directed action and action is the unit of analysis [38]. For the cases where a researcher served as a conversation partner, the parent directed the focus of the conversation and the researcher responded in ways that helped the parent talk about the weight restoration actions they were engaged in and how they engaged in these actions in their day to day lives. The joint conversation was video-recoded to captured parents’ verbal and non-verbal behaviors.

The final step of the first data gathering session was the self-confrontation procedure. Each parent was paired with a researcher to review and reflect on the playback of the video-recorded joint conversation they just completed. For Case 3, each parent separately completed the self-confrontation with a different a researcher; for cases with individual parent participants, the researcher not involved in the joint conversation conducted the video play-back. The recording was paused at regular but meaningful intervals for parents to describe their cognitions and emotions during the joint conversation, with a general guideline of stopping at one-minute intervals; parents were invited to pause the video at other times if they had something else to share.

#### First analysis

Data analysis followed the steps outlined in Young et al. [37]. All data gathered in the first session (i.e., warm-up, joint conversation, self-confrontation) was part of the initial data set, and all coding and analysis were arrived at by consensus between two researchers.

The purpose of the first analysis was to describe the actions of the parents during their joint conversation and infer their ongoing weight restoration project as manifest in the action. This analysis involved taking an iterative “bottom-up” and “top-down” perspective on the data, informed by contextual action theory. Working together, minute-by-minute, researchers read the transcriptions and re-watched the corresponding joint conversation for a three-level analysis of each minute. The first level of analysis was a bottom-up perspective that involved coding the joint conversation for specific verbal behaviours (e.g., “states a plan”, “makes a request”, “expresses disappointment”). We used a list of codes that had been developed sequentially and used in previous studies focusing on the verbal behavior of participants using this method [38, 40, 41]; Jensen and colleagues [42] discuss coding in this method extensively. The self-confrontation data corresponding to the minute provided a second level analysis of the parents’ thoughts and emotions. This data helped researchers identify the action steps (i.e., functional steps) in which parents were engaged during the action sequence (e.g., “normalizing the desire to fit into cultural beauty ideals”; “thinking about what [daughter] wants”). The bottom-up analysis focused on “why”, where asking why parents used particular elements helped reveal the function of the action and asking why they enacted a given function helped reveal their goals. Finally, the last level of analysis took a top-down perspective that provided an overall sense of social meaning that contextualized the parents’ actions. At this level, the parents’ individual and joint goals for each minute’s action sequence were identified (e.g., “to re-examine her position regarding what she wants for her daughter with the recognition she wants to consider her daughter’s wishes, too”). The top-down perspective focused on “how”, where asking how parents achieved their goals in weight restoration helped reveal the function of their actions and asking how they achieved those functions helped reveal the action elements. Researchers focused on both the how and the why in a circular way, until they achieved a clear understanding of the parent’s weight restoration actions for each minute. This circular movement between the levels in which action was embedded provided the basis for the final step of the first analysis: inferring and describing the unique weight restoration projects the parents were pursuing.

The two researchers involved in the analysis summarized the findings into a written narrative. For Cases 1, 2 and 4, one narrative was prepared for each parent. For Case 3, three written narratives were prepared: one each for the mother and father, and a third reflecting their joint action. In all cases, the narratives concluded with a tentatively identified weight restoration project. The weight restoration project was a summary statement representing the parent’s individual and joint goal-directed weight restoration actions.

#### Final data gathering session: narrative feedback & parent validation

Approximately 2 months after the first data gathering session, the written narrative and tentative weight restoration projects were shared with each participant. The purpose of this second interview was to elicit parent feedback as to whether the identified project fit their subjective experience of weight restoration, and to discuss their progress with weight restoration since the first meeting. The first author reviewed the narrative with each parent individually, and this interview was audio-recorded and transcribed as an additional source of data.

#### Final analysis

The final analysis was guided by Stake’s [39] multicase approach in which each individual parent or parental dyad became a case, and then all the cases became a basis for a multicase study. The final data set included all the data from the first analysis, plus the written narratives, the weight restoration projects, and any changes in the narratives or projects as requested by participants. Data from the final data gathering session was analyzed similar to the warm-up interview: both represented meaningful social and contextual data. The within and cross-case analysis also relied on consensus-based processes whereby the first and second authors met to discuss each case separately, identifying emerging themes, which served as the basis for the cross-cases analysis.

##### Within-case analysis

The within-case analysis was guided by the verified projects from the first analysis and sought to describe parents’ individual and joint actions while engaged in those projects. A final case summary was prepared, where the aim was to describe the complexity of the weight restoration project as it was located in its own situation. Each individual case analysis was concluded by several assertions concerning what was found to be important or salient about the parent’s actions in each case.

##### Cross-case analysis

To some extent, the cross-case analysis took place concurrent to the individual case analysis; however, full attention was not given to the cross-case analysis until the within-case analysis was completed. First, a comparison was made of how each case answered the research question. Second, significant similarities and distinctions between the weight restoration actions and processes between cases were identified, supported by quotations from the data set. Finally, key assertions were made based on the researchers’ understanding of the important findings across cases.

### Trustworthiness & rigour

Several procedures were applied to address trustworthiness in this study [43, 44]. The data set was substantial, and included manifest behavior, internal processes, and personal and social meanings, and the data was repeatedly coded, scrutinized, and discussed by the research team. Participants were directly involved in formulating and refining their weight restoration projects in the final data gathering session. Data analysis used consensus processes to heighten researcher awareness to bias related to things like selective interpretation or disregard of counterevidence. Consensus processes were also embedded in data collection (e.g., self-confrontation & parent validation of projects), as well as data coding and analysis. The self-confrontation served an important validity check on data gathered in the joint conversation, and enabled access to very specific data (and a form of triangulation) whereby parents’ personal constructions of meaning, in addition to social representations of meaning contributed by the researchers’ analytic processes, were captured and represented in the data.

## Results

The aim of this study was to describe the actions of parents as they participated in weight restoration for their adolescent. All parents shared the broad intentional framework of helping their adolescent recover weight and eliminate eating disorder behaviors, but weight restoration projects manifested themselves in unique ways in each family. Four weight restoration projects were identified in the analysis, each representing the complexity and challenges of the family in which it was enacted (see Table 3).

**Table 3:**
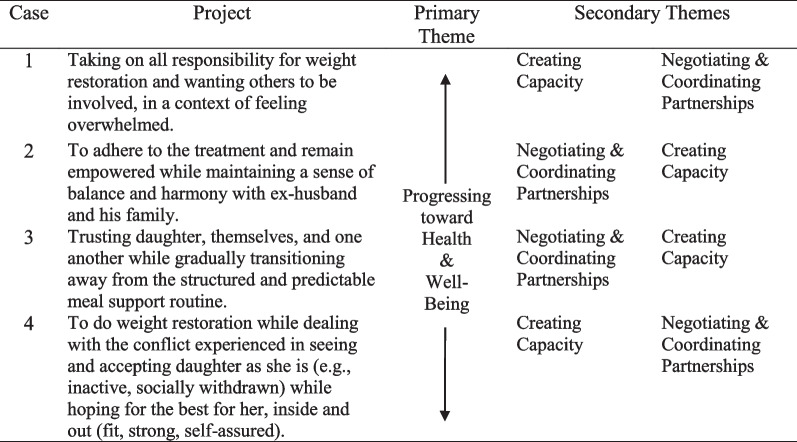
Weight restoration projects & primary and secondary themes

The order of secondary themes reflects relative salience to that case

Several properties of the weight restoration projects emerged in the analysis and were seen as important to how parents were doing weight restoration. Cross-case analysis led to the grouping of the properties into themes, where progressing toward health and well-being emerged as a primary theme, and creating capacity, and coordinating and negotiating partnerships emerged as secondary themes. The primary theme represents what the projects were principally focused on, while the secondary themes captured actions that were integral to the project, but with less prominence and varying salience given the distinct circumstances of each case. In the cross-case analysis, the socio-cultural valuing of the thin ideal emerged as a distinct process constituent to the project in Case 4. We first present the common themes and conclude with what was found to be a unique finding across the collection of cases.

### Primary theme: progressing toward health and well-being

Progressing toward health and well-being was the primary theme in all four cases, and projects focused on moving toward and attaining (a) short-term goals related to weight restoration, and (b) longer-term goals related to the adolescent’s psychological well-being. In Cases 1 and 2, weight restoration goals were foregrounded, while in Cases 3 and 4, where the adolescent was closer to reaching their minimum weight, well-being goals were more prominent. The processes by which parents progressed toward health and well-being included maintaining a holistic focus, trusting, and monitoring progress.

#### Maintaining a holistic focus

Maintaining a holistic focus on the adolescent and their development was a central process by which parents progressed toward health and well-being. All parents accepted the temporary interruption of developmental goals as a step in the service of weight restoration. Parents were motivated to do so by the treatment framework, which views anorexia as a medical crisis, but also by believing the hardship would foster resilience and work in favor of overall development (“I also know that she’ll come out of it stronger”, Case 4). However, an undulating quality of care and attention to weight and well-being goals was seen across cases. Parents’ goals in maintaining a holistic focus were two-fold: to mitigate the disruptive effects of treatment to normative development, but more centrally, to nurture growth in the psychosocial factors they believed had made their adolescent vulnerable to the eating disorder in the first place. Parents engaged in explanatory work about the cause of the eating disorder and attended to adolescent development because they viewed it as constituent to helping their adolescent recover. For example, in Case 1 the mother believed her daughter’s tendency to avoid confrontation contributed to her vulnerability in developing anorexia, and looked for opportunities to teach her daughter to ask for what she needed, even in the context of weight restoration:

She comes home from school, and goes “Mom, Dad did this at breakfast’, or you know, “I only had 5 minutes for breakfast, and we almost missed the bus’. And that’s the debate. I’ve talked to him. I’ve talked to her about-, you know, ‘You need to say that to your dad...you need to talk to your dad and tell him’. …But I still don’t know how much, and in what way, to support her. Because if I help her with it, am I teaching her? Or am I just doing it for her and then she doesn’t have to do it? And that’s-, I’m still having trouble with that, you know, as a parent.

Parents perceived the goals of weight restoration and adolescent development as competing and in tension, and they were attentive to balancing the priorities. The balancing act was distinct in each case, and actions were guided by parents’ perception of the adolescents’ progress with treatment and the specific developmental or growing edges they were trying to nurture. In most cases (1, 2, & 3), parents were cautious in tending to these growing edges and took more opportunities to do so when progress with weight was favorable (See Unique Processes, below, for how this was approached in Case 4). For example, the mother in Case 2 shared the following:

Mom: Like, what I was thinking was-, maybe because she hasn’t been gaining, we could tell her, you know “we love you, we support you in your [activity]. You can still go two times a week, but we don’t want you on the travel team this year.”
Researcher: How do you think she will respond?
Mom: I-, yeah. It all depends on her friend, of course. If her best friend is going to be able to go on the travel team, then she will be very disappointed. ‘Cause then she’d get to go and do all those fun things without her, and they want to do it together, right? Yeah. My poor dear. But you can’t have everything all at once. And I guess she might as well start learning that now

##### Trusting

Progress toward health and well-being was characterized by a gradual transition from parental trust in the treatment to trust in themselves and their adolescent. As parents progressed through the first phase of treatment, problem-solving how to step back from the structured routine of meal support and the goal of wanting to trust emerged. For instance, the mother in Case 1 shared:

And now, sort of going, “okay, where do I let go of stuff?” I don’t know. So, we’ve developed that new pattern, and that’s fine…but then I have to…there’s still a part of me that still really worries about it. Umm, but at the same time, you kind of go, “Okay, I need to, you know, trust that she is getting better and that she’ll start doing some of the stuff on her own”.

Parents approached trusting by choosing to be optimistic, but not naïve. Their motivations were varied and layered, such as wanting to be done and move on from weight restoration (Case 4), being exhausted and needing to ease their load (Case 1), knowing their adolescent longed for more normalcy (“You don’t want to say, “Okay, you can’t have friends over!’”, Case 2), and the hope that extending trust would foster respect and re-connection (Case 3). In Case 4, trusting came more easily than being cautious (“It’s hard to not trust it. And it’s hard to remember sometimes there’s mental health stuff.”), but this was not as much the case in the other families where trusting was a precarious but necessary undertaking. For example, the mother in Case 3 shared the following:

Yes, the trust issue is-, I think it’s easier and harder in some ways. Because the meal plan was-, there was so much structure. Right? It was very-, in a way, quite easy to say “Okay, this is correct. So, we do the meal plan, and it has to be-, you have to follow it 100%”. [But now] there’s situations where she calls in... “Is it okay, I’d like to stay here for dinner?” And then, you think “Uh, is it okay?”

##### Monitoring progress

Attending to markers of progress was central to progressing toward health and well-being as it revealed what was not working and needing to be changed. Also, if progress was favorable, it was a powerful motivator instilling hope the parents’ efforts were paying off. In all cases, progress was identified through direct observation of tangible markers, such as length of meals, ease of eating in new settings, and physical weight. However, progress was also inferred through monitoring less tangible markers, such as parents’ internal sense (“I notice I’m not feeling as exhausted as I did a few months ago”, Case 1), comparing behaviors to what seemed normal for a teenager, and the perceived openness and quality of connection with their daughter (all cases). In Cases 3 and 4, progress was more challenging to identify given the adolescents favorable response to treatment, and parents had to work out for themselves what these markers would look like. For example, in Case 3, the dad described using strategies like assessing how much trust he felt about his daughter’s progress and identifying what factors contributed to this trust:

Overall...um, I want to find out...how can we measure her emotional stability. How do we find out when she’s in trouble, and- and sort of, was saying yep, a couple of things we’ll be noticing, but...overall, I feel currently very good because [our daughter’s] been very open, at this time. She didn’t used to be. Never-, never in her life. And she was very, very secretive when she was in a lot of trouble. And so over the past weeks, she opened up more than ever before.

It is worth noting the two processes of trusting and monitoring progress were conceptually overlapping: Parents monitored how much trust they felt as an indicator of progress toward the overarching goal of health and well-being.

### Secondary themes

#### Creating capacity

Creating capacity refers to projects focused on resourcing the parent emotionally, personally, and practically to do weight restoration. Some of the ways parents resourced themselves included (a) learning about anorexia, FBT, and how to provide meal support (all cases); (b) partnering with others for support and assistance (all cases; see Negotiating and Coordinating Partnerships, below); and (c) finding meaning in the adversity, such gratitude for facing the challenge now rather than later (Cases 1 & 4) and the belief that going through it would foster a strong parent–child bond (Cases 1 & 3). The main processes by which parents created capacity included managing emotions to maintain a helpful focus, personal work, and resourcing time and finances.

#### Managing emotions to maintain a helpful focus

In all cases, managing emotions to maintain a helpful focus was a mainstay for creating capacity. Projects were characterized by many adaptive emotions that energized parents’ actions, such as hope, determination, devotion, gratitude, and love. But more prominent were heavy emotions that required effort to manage or resolve, such as worry, anger and frustration, guilt, uncertainty, and grief. In Case 3, co-regulation was an important strategy for managing emotions. In the joint conversation below, the dad had just revealed feeling fearful their daughter might run away. In the mom’s self-confrontation, she noted his outlook may be more pessimistic because of the different relationship they each have with their daughter (“You know, so I try to reassure him with that that I don’t worry about it, and why”). A moment later, the tables are turned where the dad senses concern in his spouse’s comment and responds with a helpful perspective:

Mom: Yeah, I’m just-, I have to say, I’m a bit nervous about her school starting.
Dad: Mhmm. [pause]. When-, remember this morning, surely you remember that, all of a sudden, she announced at the breakfast table, “it’s now 5 months...that I have not been thinking about self-, self-harming”.
Mom: No, “I haven’t done any self-harming”
Dad: “I haven’t done any self-harming”.
Mom: Five months.
Dad: That was a huge deal. For her, that was a huge deal for her to tell us. I was not aware that she’s keeping record about these things
Mom: I didn’t know either...

More evident in Cases 1, 2, and 4 were efforts to manage emotions via internal processes and self-regulation strategies. For example, managing expectations (e.g., remembering they are dealing with a mental illness, all cases), practicing acceptance of things they could not change (e.g., making peace with the eating disorder, Case 4), and learning to recognize the degree of worry they felt for their daughter might not always a trustworthy guide for action (Cases 1 & 2). The parents in Cases 1 and 2 wished there was a counselor on the treatment team assigned to support them individually.

#### Personal work

In three cases (1, 3, & 4), parents created capacity by adopting new ways of being in relation to themselves and their adolescents. For these parents, the personal work was characterized by heightened self-awareness as well as internal struggles. For example, the mother in Case 3 noted how challenges to, and the reconstruction of her self-view, helped her be successful in weight restoration:

We’ve had this role, unfortunately. You know, me-, I’m the kind one, the understanding one and [my husband] sets the rules. And he’s, you know, the disciplinarian. And it took several weeks, or months I would say, for me to understand that with- by me being kind, I was prolonging her illness. Uhm, so I had to kind of-, in a way-, really change who I was. And that was tough for me. But it was absolutely necessary.

In Case 1, maintaining a healthy weight was meaningful to the mother’s health and self-image as a breast cancer survivor. However, she had supported her daughter by eating the same high-calorie meals together, gaining weight herself overtime. Despite the discomfort she felt in her own body, and the fear her body could work against progress (“I still feel like…she looks at me and goes “yeah, I don't want to look like you…”), she choose to model body confidence as a step in service of her daughter’s weight restoration:

…You know, we just went camping and we went and did the showers together. And I’m not feeling comfortable doing it. But at the same time, going, I can’t be showing her I’m not comfortable (tearful). Because it’s about being comfortable in your skin, whatever size you are. And there’s the whole awful part around it as well (tearful), trying to teach her about…being comfortable around-, being comfortable in your own skin, but at the same time not being comfortable in mine right now (crying)”.

#### Resourcing time and finances

For Cases 1 and 2, inadequate time and financial resources presented practical challenges to the parents’ overall capacity, and for these parents, processes related to resourcing time and finances were fundamental to their projects. For example, strategizing ways to manage work absences given lost income and depleted savings featured prominently in these cases. Functional steps here included things such as driving a longer distance to get cheaper groceries, cutting back on sleep to fulfill work obligations, turning down new work despite needing the income, and reluctantly asking older children to monitor meals that could not be supervised due to work. In the months ahead of the interview, the mother in Case 2 lived off her savings and a medical leave to care for her daughter. A day before the interview, she learned her daughter had lost 2 pounds, a setback that made the challenge of resourcing weight restoration feel insurmountable:

Now, what if it happens again, I have-, I can’t take anytime off work, I don’t have any money, I don’t know how I’m going to help her!” …Like, I’m done. I’m done. I’ve done everything I can. And, all my cards are out, and gone.

##### Negotiating and coordinating partnerships

Negotiating and coordinating partnerships refers to projects that were focused on staying united in their efforts to ensure effectiveness (e.g., meal support, parental decisions about trust, family messages about fitness and health). The projects were characterized by many kinds of partnerships, including professionals, older children, and extended family members, but the central partnership being coordinated and negotiated was with the co-parent. An array of family structures and relational dynamics were represented in the data, and the process of negotiating and coordinating with the co-parent was distinct in each case. For example, in Case 3, the partnership was approached through functional steps like sharing concerns and brainstorming solutions, dividing roles and responsibilities, and monitoring for agreement and consistency in plans and actions over time. During the joint conversation, the dad noticed his spouse had been worrying about something and had not included him in trying to address it. He shared the following in his self-confrontation:

Dad: Um, so, if she’s so worried about [our daughter], what [she] would have for breakfast and lunch, why didn’t she tell me that? She sort of already had a plan.
Researcher: You wanted to be included.
Dad: I wanted to be included. That was-, for us-, that was very clear. Because we sometimes were on different pages, and sometimes we didn’t agree. We needed to agree, and the best way to do it is to sort of put out a plan. Whether it’s written or not. Let’s say, “Okay, this is what I think, this is what you think, do we agree?”

The mother in Case 1 also monitored her own and her spouse’s actions for agreement and consistency. However, his low-level of involvement created additional problems to manage and solve and she believed doing weight restoration on her own would have been easier. Although the mother had accepted full responsibly for weight restoration, coordinating with her spouse remained central given the need to ease her burden. Functional steps in this case included processes like updating him on what was difficult for their daughter at mealtimes, asking him to say things that would be supportive and helpful to the daughter, and dealing with his anger and irritation at being asked to change. Emotional processes salient here included feeling let down and abandoned, and wishing for her efforts and sacrifices to be acknowledged and appreciated:

And so much of that frustration with- with him not...I keep saying, him not buying in, but him not, like, just wanting to find out “what can I do to help?” I've never heard him say that. I've never heard him say “What do you need me to do to help?" or “How can I help?" "What do I need to change to help [our daughter]?" It's all like-, it's all like, getting mad at me for forcing him to do something...

The projects in Cases 2 and 4 were negotiated and coordinated in the context of a divorce and separation, where the superordinate goals were (a) to pull together with their co-parent for their adolescent and (b) to preserve the adolescents’ sense of unified family support. Choosing their battles carefully and practicing acceptance of disparate parenting philosophies and weight restoration strategies were salient functional steps in both cases. At the level of emotions, the mothers felt gratitude for the involvement of the co-parent but also worried about introducing conflict that would strain the relationship and make things more difficult. For example, in Case 2, the mother hoped meals would happen similarly at both homes but was reluctant to act toward this goal:

Mom: I should be asking them more [about meals], but I don’t, I don’t want...to be a bitch either, you know?
Researcher: Do you feel like you would be being a bitch if you did that? Or that they would interpret it that way?
Mom: Yeah, they would definitely interpret it that way. Yes. I don’t know how to explain it. And it’s probably just my interpretation of it all, and who knows if I’m right. But it’s uncomfortable if you make anything uncomfortable for them [laughs]. They make it double or triple uncomfortable for you. So basically, I just don’t go there.

The partnership was negotiated by preserving harmony between the homes, which in this case meant not knowing how well coordinated the efforts were:

It just adds more worry to me because, I don’t know, I just wish he would take it more seriously. Sometimes I think that he just doesn’t take it as seriously as-, because my set schedule, I don’t think they do that at their house.

The partnership in Case 4 was negotiated by resuming old family routines and coping with the emotions, as well as old and new conflicts, this stirred up. For example, on resuming family dinner-time meals shortly after their daughter’s diagnosis, the mother shared:

At first it was like, “Oh, you still want me to cook for you?” Like, literally. “Oh you don’t want to come over on the weekends, but you’re okay during the week, after you’re home from work and you’re tired, I feed you?” Like, “Hello? Uh, that’s not happening”. And then, you know, it’s like, “Actually, we need to do this for [our daughter]. Darn. Okay”. So suck it up, bury those emotions.

### Unique processes

A unique process in the collection of cases emerged in Case 4, where the project arose from and was enacted within a belief system that viewed a slim body to confer protective factors for the adolescent: “the thinner you are, the more beautiful you are, the easier the world is for you. Like I-, I truly…believe that. You know, they’ve done studies on it”. In this case, the mother’s idea of well-being rested heavily on social acceptance: “You want your kid to be-….to fit in, to have awesome friends, and obviously that’s easier if you look a certain way as well”. The tension between weight and well-being goals was resolved by the mother approaching weight restoration with healthy foods with the hope the daughter would not exceed her ideal weight. These underlying beliefs and actions also shaped the parent’s goals and actions in relation to the treatment:

Researcher: But there’s some aspects of the re-feeding that created some uneasiness?
Mom: Oh, totally. Yah. A lot of uneasiness. For her, and I’d say myself as well. What if they’re wrong? What if they make her gain too much weigh, and then- and then she feels like she’s…too heavy.
Researcher: And that might keep her from going out and being social?
Mom: Yeah. Yeah. You’ve hit it-, I think you’ve hit it really well. It’s sad, it’s scary.

The mother’s examination of her own beliefs in relation to cultural ideals related to body shape, self-esteem, and social acceptance was an ongoing process, as evidenced by the following from her self-confrontation:

Uhm, yeah. Just trying to put pieces together. Being kind of confused myself. How do I express what I think she might be going through? As I’m watching it back, I’m thinking, those are-, those are my thoughts, and are those my confusions. Maybe my issue more than her issue? My issue for her?

## Discussion

This study described how parents participated in the weight restoration of their adolescent while engaged in FBT for anorexia. It is the first study to examine parents’ actions while they implemented the treatment and represents an important shift in thinking from discrete parent or treatment variables correlated to or predictive of weight outcomes [45, 46] to the processes, properties, and systems through which parents enact weight restoration. The parents’ weight restoration projects were decidedly intertwined with other significant and pre-existing projects and careers, for example, parenting, identity, occupation, and marriage. The complexity of these interconnections was reflected in their actions. Many actions were undertaken individually by parents, but their actions in weight restoration did not stand alone: Parents acted jointly with their co-parent, their adolescent, and treatment professionals relative to the goals of weight restoration. This is a vastly expanded view of parents’ participation in FBT, and the goal-directed perspective adds to the literature by describing how some parents translated the principles of FBT into their day to day lives. In this study, parents participated in weight restoration by progressing toward health and well-being, creating capacity, and coordinating and negotiating partnerships.

### Progressing toward health and well-being

The main processes by which parents in this study progressed toward health and well-being included maintaining a holistic focus, monitoring progress, and trusting. Parents acted as agents of change vis-à-vis the treatment goals and did so in service of a broader parenting project concerned with the adolescent’s overall well-being. For the parents in this study, weight restoration and adolescent development were enacted simultaneously with reciprocal influence well before the adolescent achieved a stable weight, as is recommended in FBT. Some parents navigated the tensions and balanced the competing priorities by trusting the treatment framework, finding meaning in the adversity, as well as referencing their informal theories about the cause of the eating disorder to guide day-to-day decision making. Explanatory work is viewed as an impediment in FBT because it risks producing parental guilt, causing self-doubt, hesitancy, and ineffectiveness [1]. However, parents’ informal theories seemed to offer a sense of coherence [47] as well as a blueprint for supporting their adolescents’ overall recovery. The premise that explanatory work is an impediment to treatment and that adolescent development is stopped by anorexia are strategic elements in FBT designed to motivate parental action. While these premises may help set the stage for action initially, practitioners may find it helpful to understand this is quite removed from the lived reality of parents, who engaged in meaning making with a purpose, and continued to relate to their adolescents as whole persons, complex and multifaceted. This latter finding aligns with research on the adolescents’ perspectives of treatment for AN, who believe treatment must consider their unique characteristics and social circumstances [48, 49].

Monitoring progress and trusting were the main functional steps parents took in service of progressing toward health and well-being. In monitoring progress, parents looked to the adolescent’s physical weight but also assessed less tangible markers to evaluate the effectiveness of their efforts, such as the quality of their relationship with their adolescent. With evidence of steady and reliable progress, the challenge of trusting emerged for parents. Trusting was the process by which parents moved away from the structured routines of treatment, and their motivations to trust were varied and layered. For example, parents were motivated by hope, exhaustion, and necessity, as well as successful weight gain. In FBT, progress is marked by the gradual return of parental control over food and weight back to the adolescent [1]. However, the perception that progress is characterized by a return of control may better reflect the perspective of professionals whose theory of change rests on parents taking control of the adolescents’ eating. Practitioners’ may find broadening conceptualizations of progress to include trust-related processes better reflects how parents experience and explain their own actions.

### Creating capacity

Increasing parental self-efficacy with the tenets of FBT is the main approach to mobilizing parental resources [4, 19, 50]. In this study, the actions and processes by which parents resourced weight restoration were illustrated. Parents created capacity through actions such as researching and planning, problem-solving, collaborating with others, and regulating their emotions, attitudes, and behaviors. For some parents, enacting weight restoration challenged self-perceptions, disrupting and sometimes revising their identities. Hoskins and Lam [51] previously found the experience of mothering a child with anorexia set in motion a protracted process of identity redefinition, and Bezance and Holliday [52] identified a diminished sense of identity as parents were consumed with their daughter’s needs to the neglect of their own. The current study extends these findings to FBT, showing some parents intentionally generated, re-constructed, and worked to maintain self-systems in order to guide and resource their actions. The self-reflective and self-regulative processes by which parents created capacity to enact weight restoration have not previously been addressed in the literature.

Parents in this study were both the producers of resources, and their actions were shaped by the resources available. Some of the greatest challenges faced by parents were because they lacked resources, including unmet financial, social, counseling, or psychoeducational needs. Poorly resourced projects created practical and emotional challenges that diverted parental energy and attention away from the steps of treatment. Hillege et al. [53] and Keitel et al. [54] found a scarcity of resources, especially financial and social, contributed significantly to the challenges of caring for a child with anorexia, and clinicians treating families with FBT have previously observed “poor parents [can’t] do it” ([24] p549). The findings of this study indicate there may be a structural basis for some parents’ struggles whereby they are disadvantaged in realizing favorable treatment outcomes. Sociostructural and other contextual variables have remained ignored in much of the psychological treatment literature [55], including FBT (e.g., [3]). Recognizing the role of such factors merits increased attention within eating disorder research generally, and FBT specifically. The families who participate in FBT in community will have a greater diversity in ethnicity, social class, and family composition and structure than families in clinical trials. Goal-oriented frameworks may help practitioners stay mindful of the ways parents’ actions are shaped by sociostructural elements and reveal opportunities for advocacy work or other adaptations to help reduce barriers to success.

### Coordinating & negotiating partnerships

FBT was developed on the premise that two parents from an intact family would be available to help facilitate the treatment and stresses the importance of parental unity to treatment success [1, 17]. Two studies comparing outcomes between single and dual parent households were located and confirmed FBT can be equally efficacious for different family structures [56], where some nonintact families may benefit from longer treatment [57]. However, the findings of this study support Celio-Doyle et al.’s [58] observation that family structure alone may be a poor proxy for the pertinent elements of family context that influence treatment outcomes, including how parents share responsibilities, the nature of family resources, or how united parents are. These findings confirm weight restoration takes place in relational systems that shaped, constrained, and in some cases, blocked parents’ efforts. Parents contending with a distant, uninvolved, or separated co-parent, whether they lived together or not, faced challenges that complicated their efforts and brought about difficult emotional dimensions to their projects. In such cases, several actions and processes over-and-above those of the treatment framework became necessary, such as petitioning for the co-parent’s involvement and support, attempting to influence a co-parent’s actions or attitudes, and accepting and tolerating a co-parent’s disparate views or strategies. Many augmentative approaches to improve treatment outcomes in FBT use adjunctive therapeutic components, such as emotionally focused family therapy [19], multifamily therapy [59], cognitive behavioral therapy [60] or dialectical behavioral therapy [61, 62], but such approaches have not yet been extended to supporting the parental dyad. The pivotal role parents play points to fostering or restoring parental unity as a tangible and practical way to favorably influence treatment outcomes. An adjunctive intervention for parents struggling with unity seems a viable treatment adaptation unlikely to interfere with the core elements of FBT.

### Limitations

This study could have been strengthened in a variety of ways. First, the sample of participants had limited diversity as most were mothers who identified as Caucasian. It is reasonable to expect the actions of parents from diverse ethnic and cultural backgrounds may be distinct and diverge in important ways from those described by parents here. In addition, the underrepresentation of fathers is an important limitation not uncommon to research on caregivers’ experiences of anorexia. Considering how prominently current and ex-partners featured in the actions of mothers in this study, it would have been informative had their voices been captured in the findings. Second, in real life a parent’s weight-restoration actions would take place constantly in many contexts, but issues such as privacy and practicality make access to these actions difficult. Action-project method was developed with this challenge in mind, and the method looks at interactions and dialogue between a participant dyad thereby allowing for data on joint action. For the single parent participants, researchers acted as conversation partners to create a dyad. Given the relational nature of weight restoration, it is reasonable to expect the absence of this data diminished the richness and fullness of the findings.

## Conclusion

This study sought to increase knowledge and understanding of parent-led weight restoration by investigating it from the parent’s perspective during the time they were engaged in these actions. It examined parents’ actions through the perspective of contextual action theory, attending to parents’ actions in context and bringing the voice of the parent to the forefront. The findings supplement and extend current thinking about how parents achieve weight recovery goals in FBT and shows that in addition to applying behavioral strategies to bring about change, parents undertake a myriad of explicit and tacit goal-directed actions and strategies that are embedded in and emerged from a larger system of intertwined projects and careers in their lives. As parental involvement is elemental to favorable treatment outcomes in FBT, it is hoped the study findings will inspire ongoing efforts to assist and support parents engaged in this treatment.

## Data Availability

The dataset used during the current study are available from the corresponding author upon reasonable request.

## Abbreviations

FBT: Family based treatment
